# Evolution of ZnO Nanorods from Faceted Crystals to Near-Spherical Nanoparticles Under Controlled Laser Irradiation

**DOI:** 10.3390/nano16181146

**Published:** 2026-09-13

**Authors:** Muidh Alheshibri

**Affiliations:** General Studies Department, Jubail Industrial College, P.O. Box 10099, Jubail Industrial City 31961, Saudi Arabia; alheshibri.muidh@gmail.com or heshibri@rcjy.edu.sa

**Keywords:** metal oxide nanoparticles, zinc oxide, laser irradiation, morphology, nanomaterials

## Abstract

Owing to its favorable physical and chemical characteristics, zinc oxide (ZnO) has become one of the most extensively investigated metal oxide semiconductors. Pulsed laser irradiation in liquids provides a post-synthesis route for modifying ZnO nanostructures. However, correlating laser parameters with the systematic evolution of particle morphology, size distribution, and surface chemistry remains challenging. In this work, a facile and additive-free pulsed laser irradiation approach was employed to engineer the morphology and surface chemical composition of ZnO nanoparticles. As-prepared hydrothermally synthesized ZnO nanorods dispersed in deionized water were irradiated with a Q-switched Nd:YAG laser for 30 and 90 min and compared against the untreated powder. TEM and SEM analyses revealed a progressive morphological transformation in which the strongly agglomerated, faceted nanorods partially reshape into a mixed rod and particle morphology after 30 min and evolve into well-dispersed, nearly spherical nanoparticles with diameters of approximately 12–48 nm after 90 min. XRD confirmed that the hexagonal wurtzite structure is retained throughout the treatment with no secondary or impurity phases, while the mean crystallite size increases from 15.14 nm for the powder to 18.80 nm after 90 min of irradiation as a result of photothermal fusion. XPS demonstrated that zinc preserves its +2 oxidation state during laser processing, whereas the surface O/Zn ratio and the relative fractions of lattice oxygen and hydroxyl species evolve systematically with irradiation time, revealing a concurrent modification of the surface chemistry. These results establish the laser post-irradiation duration as a simple and effective control parameter for tailoring both the morphology and surface chemical state of ZnO nanostructures.

## 1. Introduction

Zinc oxide (ZnO) has attracted considerable attention among metal oxide semiconductors owing to its combination of physical and chemical properties. It possesses a wide direct band gap of ~3.37 eV together with a large exciton binding energy of about 60 meV, which remains stable at room temperature [1]. ZnO is a pivotal material in optoelectronic devices due to its unique combination of intrinsic electronic, optical, and structural properties [2,3]. ZnO also possesses piezoelectric behavior [4], high chemical stability [5], low toxicity [6], and earth abundance [7], which collectively extend its applicability across a broad spectrum of fields including gas sensing, photocatalysis, solar cells, biomedical devices, and energy harvesting [8,9,10]. The reactivity of ZnO is considerably enhanced at the nanoscale due to quantum confinement and the increase in surface-to-volume ratio [11].

The behavior of nanosized ZnO depends largely on its morphology. The literature contains different synthesized forms of ZnO including nanoparticles, nanorods, nanowires, nanoflowers, nanosheets, nanobelts, and hollow nanospheres, among many others [3,12]. This morphological diversity arises from the anisotropic growth rates of distinct crystallographic facets and the sensitivity of surface energies to reaction environment, temperature, pH, and capping agents [13,14,15]. The surface chemical composition, quantified through the relative concentrations of lattice oxygen, surface hydroxyl groups, and oxygen-vacancy-associated species, is itself a function of morphology, as differently faceted surfaces present distinct atomic environments with different surface energies and reactivities [16,17]. Thus, the ability to deliberately engineer ZnO morphology represents a powerful method for optimizing the material’s application-relevant properties. A range of methods has been established for this purpose, including hydrothermal methods, chemical vapor deposition, sol–gel processing, microwave-assisted aqueous routes, and solvothermal synthesis [9,18,19]. However, these methods often demand elevated temperatures, extended reaction times, complex precursor chemistries, or chemical additives that introduce surface contamination and constrain post-synthesis processability. Thus, there is a need to develop alternative approaches capable of engineering ZnO morphology in a controlled, additive-free manner.

Pulsed lasers with pulse durations ranging from femtoseconds to nanoseconds play distinct roles in advanced manufacturing [20,21]. This technique has emerged as a powerful and versatile tool for the synthesis of colloidal nanomaterials. It offers key advantages over conventional wet chemical methods [22,23,24,25]. In pulsed laser ablation, a focused nanosecond, picosecond, or femtosecond laser pulse is directed onto a solid target immersed in a liquid medium, inducing rapid ablation, plasma formation, and cavitation bubble dynamics that collectively drive nanoparticle nucleation and growth at the solid–liquid interface [23,26,27]. The technique produces highly pure nanoparticles free of chemical reducing agents, capping ligands, or organic solvent. This purity is particularly advantageous for biological and catalytic applications where surface contamination must be minimized [28,29]. Laser parameters including pulse energy, repetition rate, pulse width, wavelength, and ablation time, as well as the nature and composition of the liquid medium, collectively determine the size, morphology, and surface chemistry of the resulting nanoparticles [30]. Among these variables, secondary laser irradiation of the as-produced colloidal suspension has received growing attention as a post-synthesis strategy for reshaping nanoparticle morphology without altering chemical composition. When a colloidal suspension of nanoparticles is irradiated with a laser beam, photon energy is absorbed by the particles, raising their local temperature through a photothermal mechanism. Depending on the laser fluence and absorption cross-section of the particles, this heating can induce partial or complete melting, fragmentation, surface restructuring, and Ostwald ripening, all of which drive morphological transformation [30]. For instance, colloidal ZnO nanoparticles irradiated at 355 nm have been shown to undergo transformation from nanorods to nanosheets through photothermal melting and fusion [28]. Drmosh et al. employed nanosecond pulsed laser ablation (355 nm, 10 Hz, 8 ns) to change the morphology of ZnO nanoblocks (~434 × 104 × 236 nm) into thin nanosheets with a larger surface area, supported on reduced graphene oxide [31]. The resulting ZnO/rGO structure was subsequently decorated with gold nanoparticles. This laser-induced morphological transformation increased the available surface area and contributed to improved hydrogen sensing under UV illumination at room temperature. The irradiation duration is a critical parameter here, since it sets the cumulative energy dose delivered to the suspension and hence the extent of the transformation [32]. Laser post-irradiation also modifies the surface chemical composition of ZnO, changing the relative concentrations of lattice oxygen and defect-associated oxygen species as resolved by XPS [33]. This chemical modification was accompanied by a gradual morphological transformation toward predominantly spherical nanoparticles as the pulsed laser irradiation time increased. Further, the bandgap was found to be decreasing with prolonged irradiation. These changes improved the visible-light photocatalytic performance for the degradation of organic dyes [33].

Earlier studies have revealed that laser pulse irradiation in liquid can significantly alter morphology, size, and physicochemical properties of ZnO nanomaterials [31]. However, a direct correlation between irradiation time, morphology, and size distribution, along with the corresponding surface chemistry, remains less explained. In the present work, we adopt a facile pulsed laser irradiation approach to systematically engineer the morphology and surface chemical composition of ZnO nanoparticles through controlled post-irradiation. ZnO nanorod powder was exposed to nanosecond pulsed laser irradiation for 30 and 90 min and compared with the untreated reference. Progressive irradiation drives a notable transformation of the material. The agglomerated, sharply faceted crystals of the pristine powder evolve into dispersed, irregularly shaped rods and particles at an intermediate stage, and ultimately into predominantly rounded, near-spherical nanoparticles with a narrowed size distribution. This morphological reorganization is accompanied by a parallel evolution of surface chemistry, resolved here through a complementary set of characterization techniques including XRD, XPS, TEM, and SEM.

## 2. Materials and Methods

### 2.1. Synthesis of ZnO Nanorods

All chemicals used in this study were obtained from Sigma-Aldrich (St. Louis, MO, USA). A total of 3.00 g of zinc nitrate hexahydrate (Zn(NO_3_)_2_·6H_2_O) together with 4 g of sodium hydroxide (NaOH) were dissolved in a mixture of 5 mL deionized water and 10 mL diethylamine. The solution was sonicated for about 30 min, with the pH maintained at 13 throughout. It was then transferred to a Teflon-lined autoclave and held at 190 °C for 60 min under hydrothermal conditions. The resulting nanosuspension was washed several times with distilled ethanol and deionized water and finally dried at 70 °C for 24 h.

### 2.2. Laser Post-Irradiation of ZnO Nanorods

**Figure 1:**
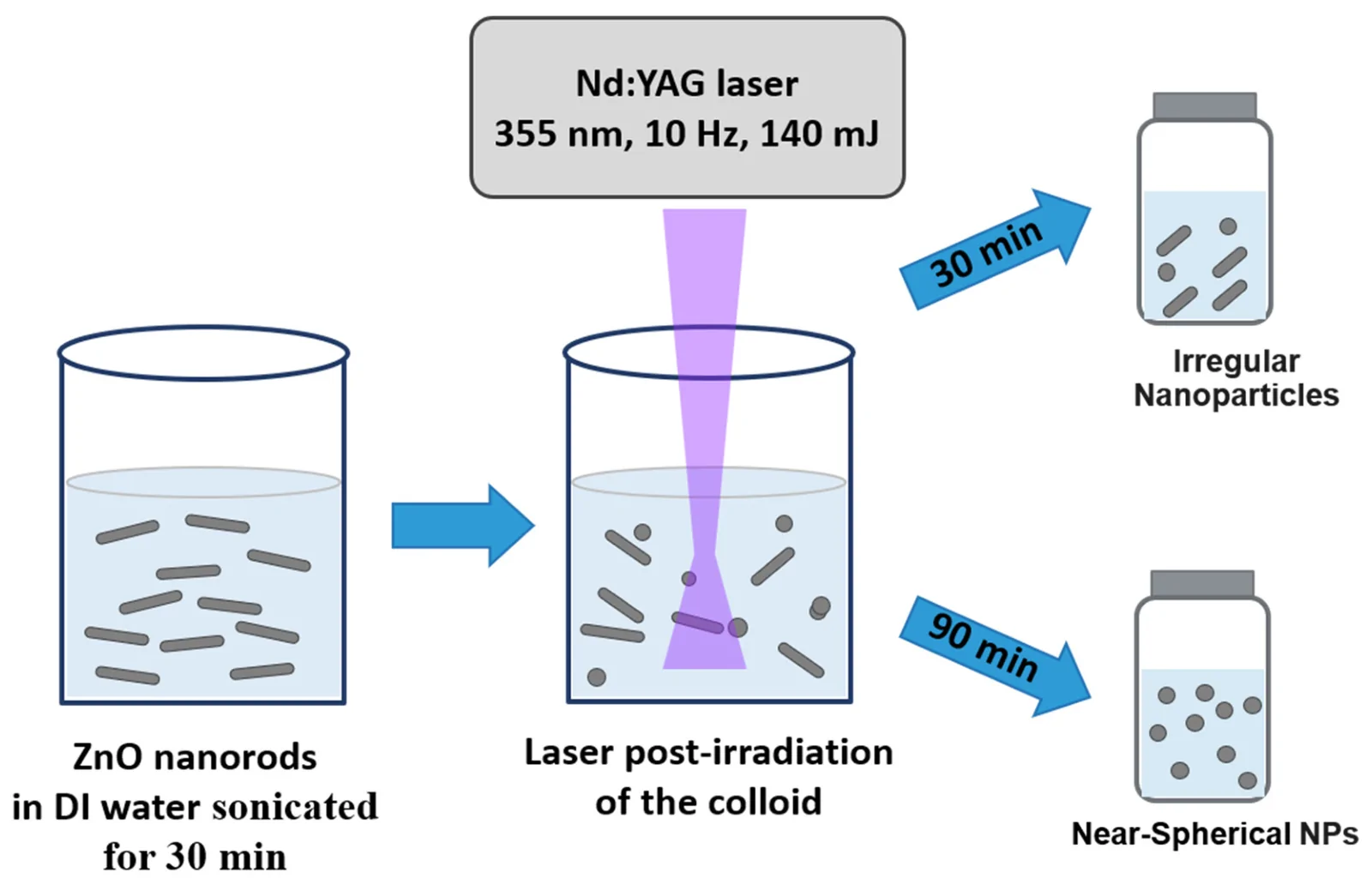
Schematic illustration of ZnO Nanoparticles fabrication.

A total of 10 mg of the as-synthesized ZnO nanorod powder was placed at the bottom of a glass vessel filled with 7 mL of deionized water. The suspension was sonicated for 30 min before irradiation. A Q-switched Nd:YAG pulsed laser operating at a wavelength of 355 nm, a repetition rate of 10 Hz, a pulse energy of 140 mJ, and a pulse width of 10 ns was used as the irradiation source (Figure 1). The colloidal nanoparticles were irradiated at room temperature with a laser fluence of ≈0.50 J/cm^2^ per pulse and a peak power density of ≈5 × 10^7^ W/cm^2^.

During the irradiation process, continuous magnetic stirring was applied to ensure uniform exposure of the nanorod powder to the laser beam. Two irradiation durations were employed, namely 30 and 90 min, to systematically investigate the effect of laser exposure time on the morphology and surface chemical composition of the irradiated material. The as-prepared ZnO nanorod powder, prior to any laser treatment, was retained as a reference sample. All irradiated samples were subsequently collected and stored under ambient conditions for further characterization. No unreacted or oversized particles were removed at any stage.

### 2.3. Characterization Techniques

The morphology of the samples was investigated using scanning electron microscopy (SEM) (TESCAN VEGA COMPACT, 30 kV, SE detector, TESCAN GROUP a.s., Brno, Czech Republic) and transmission electron microscopy (TEM). To prepare SEM samples, a drop of each suspension was deposited onto an aluminum stub covered with conducting carbon tape, dried in air, and coated with an ultra-thin gold layer to minimize charging effects prior to imaging. TEM samples were prepared by depositing a small droplet of the suspension onto lacy-carbon-coated grids with micro/nano-holes and allowing it to air-dry prior to examination. An aliquot of the as-irradiated suspension was drop-cast directly onto the cleaned substrate and dried for the remaining characterization. The crystal structure was determined by X-ray diffraction (XRD) using a Rigaku Ultima IV diffractometer (Rigaku Corporation, Tokyo, Japan) with CuKα radiation (λ = 1.5406 Å) over a 2θ range of 20–90° at a scanning rate of 0.02°/min. The mean crystallite size was estimated using the Scherrer equation. The surface chemical composition was analyzed by X-ray photoelectron spectroscopy (XPS) using a Thermo Fisher Scientific instrument (Waltham, MA, USA), where peak fitting was performed with CasaXPS software version 2.0.71 and all binding energies were referenced to the C 1s adventitious carbon peak at 284.8 eV. All three samples were characterized under identical experimental conditions.

## 3. Results and Discussion

**Figure 2:**
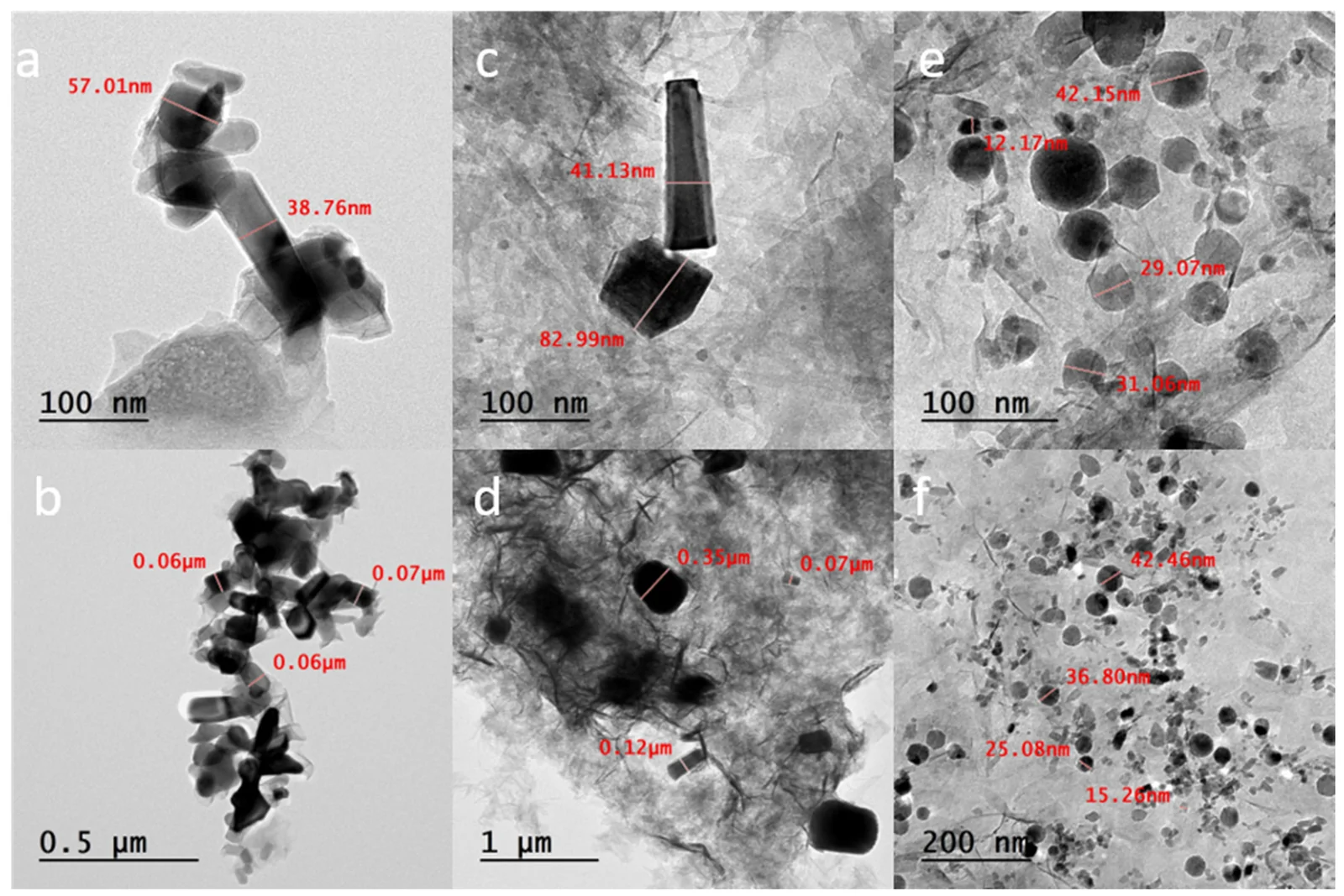
TEM images of the ZnO nanostructures: (**a**,**b**) high- and low-magnification images of the as-prepared ZnO powder, (**c**,**d**) after 30 min of laser irradiation, (**e**,**f**) after 90 min of laser irradiation.

**Figure 3:**
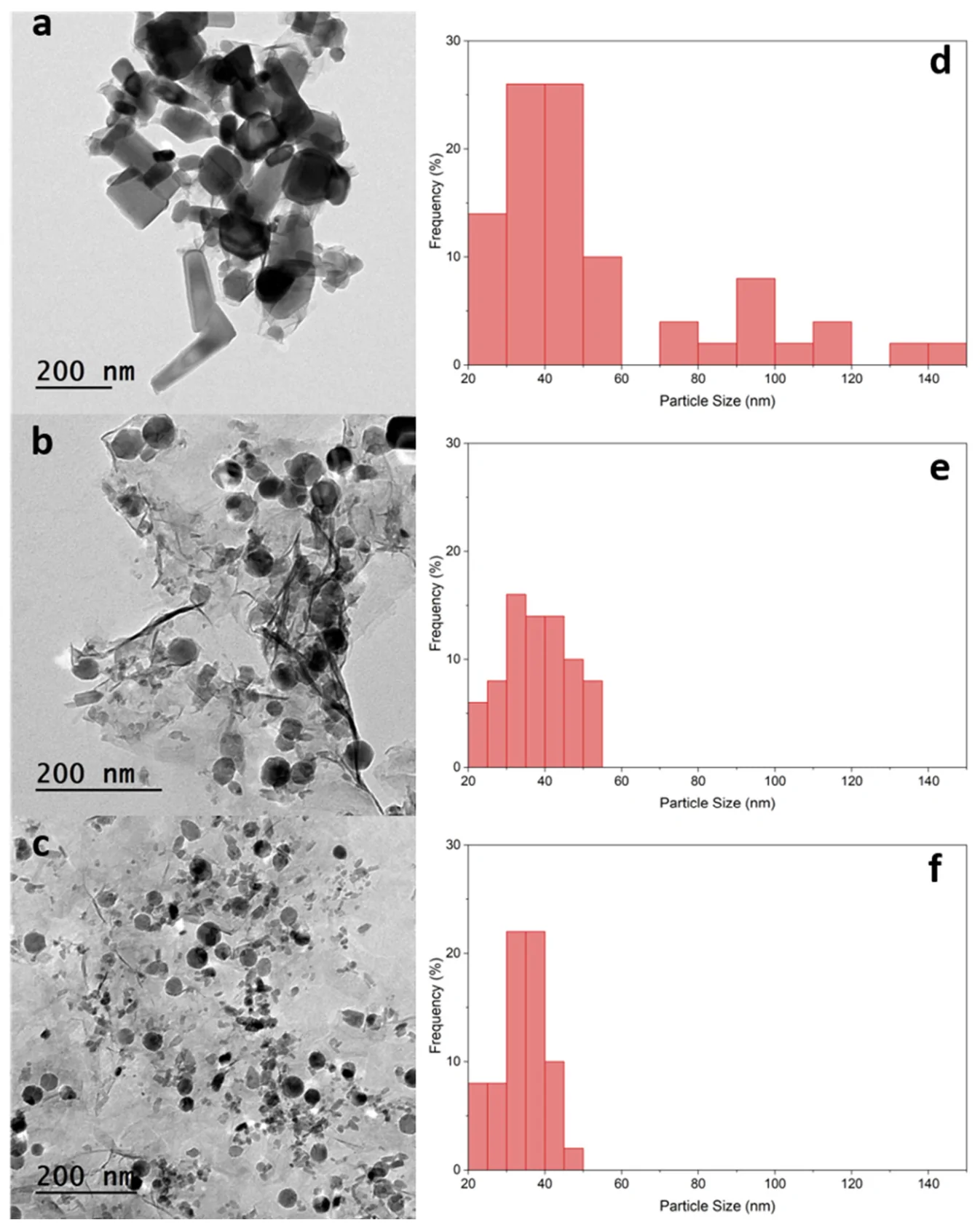
TEM images (**a**–**c**) and corresponding particle size distribution histograms (**d**–**f**) of ZnO nanoparticles at 0, 30, and 90 min irradiation time.

The internal fine structure and particle size evolution of the ZnO nanostructures before and after pulsed laser irradiation were examined by TEM, and the results are presented in Figure 2 and Figure 3. Figure 2a,b shows high- and low-magnification TEM images of the as-prepared ZnO powder, respectively. The as-prepared sample consists of well-defined hexagonal nanorods with smooth and sharply faceted side walls. A relatively high degree of agglomeration is observed in the sample.

After 30 min of laser irradiation (Figure 2c,d), the sample exhibits a mixed morphology in which the original nanorods coexist with newly formed nanoparticles. The low-magnification image (Figure 2d) shows partially fragmented agglomerates together with dispersed particles, suggesting that the absorbed laser energy simultaneously breaks down the large agglomerates and initiates the reshaping of the individual rods. It should be noted that the faint sheet-like contrast observed in the background of Figure 2c–f originates from the lacey carbon support film of the TEM grid and does not correspond to the ZnO sample.

Extending the irradiation time to 90 min (Figure 2e,f) results in the near-complete disappearance of the rod-shaped morphology and the formation of nearly spherical ZnO nanoparticles. The high-magnification image (Figure 2e) shows quasi-spherical particles with smooth, rounded outlines, while the low-magnification image (Figure 2f) confirms that this transformation occurs over the entire sample, with a large population of well-dispersed nanoparticles. In addition to the shape transformation, the 90 min sample shows a markedly reduced degree of agglomeration compared with the as-prepared powder, indicating that prolonged laser irradiation also promotes the disaggregation of the ZnO nanostructures.

To evaluate the morphological evolution induced by laser irradiation, the size distributions of approximately 50 particles were measured from TEM micrographs for each condition, and the respective size distributions are presented in Figure 3. The pristine ZnO powder demonstrated a broad size distribution, with a mean size of 54.80 ± 30.88 nm and values ranging from 23.82 to 149.55 nm. After 30 min of laser irradiation, the mean size decreased to 32.53 ± 13.10 nm, with a narrower distribution ranging from 11.08 to 53.98 nm. A minority of larger, unfragmented rod-like particles (up to ~83 nm) were still occasionally observed at this stage (see Figure 2c), indicating that fragmentation was not yet complete after 30 min. Following 90 min of irradiation, the mean size further decreased to 29.22 ± 9.60 nm, with values ranging from 12.77 to 48.06 nm. These results demonstrate a progressive reduction in the measured particle dimensions and narrowing of the size distribution with increasing irradiation time, consistent with the morphological evolution observed in the TEM images.

The observed morphological evolution may be associated with photothermal interaction between the 355 nm laser pulses and the ZnO nanostructures. The interaction of nanosecond laser pulses with colloidal nanoparticles is greatly influenced by the balance between the energy a particle absorbs and the energy needed to heat, melt, and evaporate it [34]. Since the photon energy at 355 nm (3.49 eV) exceeds the band gap of ZnO (~3.37 eV), optical excitation of the ZnO nanostructures can occur. Under the applied laser fluence (~0.50 J cm^−2^ per pulse), absorption of the laser energy may generate transient localized heating, promoting morphological restructuring and fragmentation. The applied laser fluence in this study (~0.50 J/cm^2^ per pulse) is well above the critical fluences reported for both complete melting and evaporation of ZnO nanoparticles [35], indicating that the particles can receive sufficient energy to undergo substantial thermal modification during laser irradiation. If sufficient local heating occurs, surface energy minimization may favor spheroidization of the initially anisotropic structures, while higher absorbed energies may promote fragmentation into smaller particles [27,36]. For nanosecond laser irradiation, melting and fragmentation have been reported to occur simultaneously, with their relative contributions depending on the absorbed energy [36]. Such processes are consistent with the coexistence of residual nanorods and newly formed nanoparticles observed after 30 min of irradiation. Similar laser-induced shape transformations from anisotropic to spherical morphologies have been widely reported for metal and metal oxide nanostructures subjected to pulsed laser irradiation in liquid media [27,30,36].

**Figure 4:**
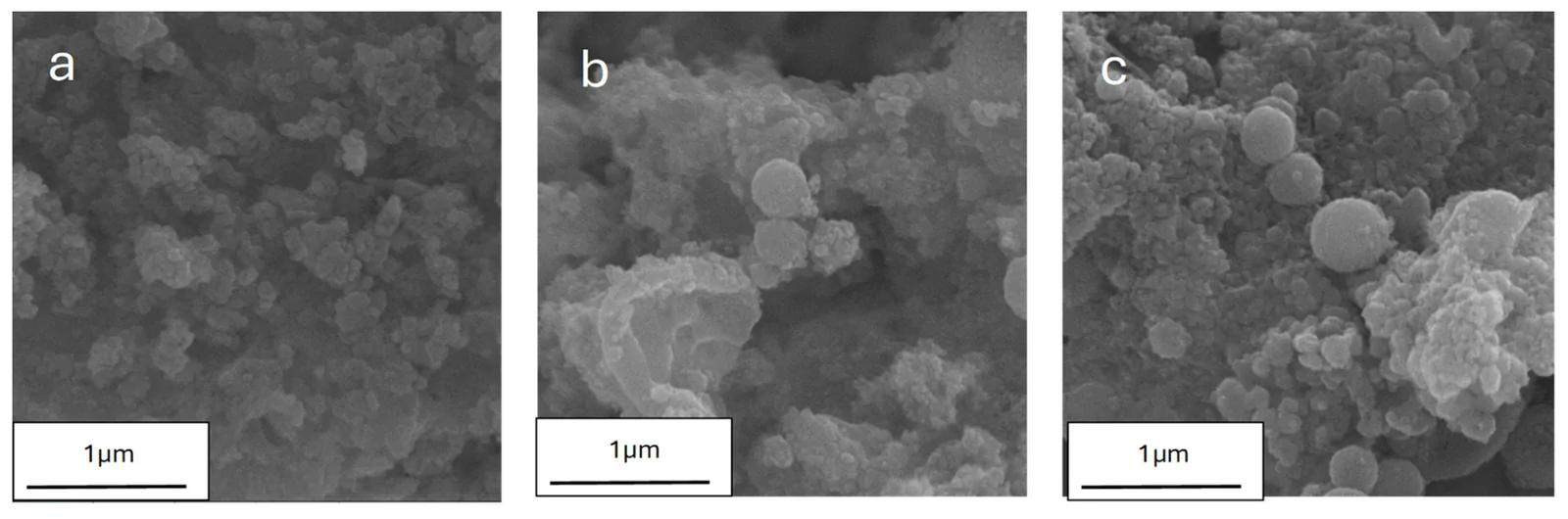
SEM images of the ZnO nanostructures: (**a**) as-prepared ZnO powder, (**b**) after 30 min of laser irradiation, (**c**) after 90 min of laser irradiation.

While the TEM analysis established the morphological transformation of the ZnO nanostructures at the level of individual particles, SEM was employed to examine whether this transformation proceeds uniformly over large areas of the sample. Figure 4 presents SEM images of the as-prepared ZnO powder and the samples irradiated for 30 and 90 min. The as-prepared powder (Figure 4a) consists of densely packed ZnO nanorods with well-defined, faceted side surfaces, which are strongly agglomerated into large, compact clusters.

After 30 min of laser irradiation (Figure 4b), the sample exhibits a clearly mixed morphology in which residual nanorods coexist with newly formed, rounded nanoparticles distributed across the imaged area. The compact agglomerates of the as-prepared powder appear visibly loosened, and a few individual nanostructures become more distinguishable, indicating that the absorbed laser energy begins to promote the break-up of the large clusters in parallel with the reshaping of the rods.

Following 90 min of irradiation (Figure 4c), the rod-shaped morphology has disappeared entirely and the sample surface displays a homogeneous granular texture composed of nearly spherical nanoparticles. No residual rods or faceted fragments could be detected in any of the examined regions. The 90 min sample also shows a markedly more open, less agglomerated packing than the as-prepared powder, in agreement with the disaggregation observed by TEM and with the general tendency of pulsed laser irradiation in liquid media to simultaneously reshape suspended nanostructures [27,36].

**Figure 5:**
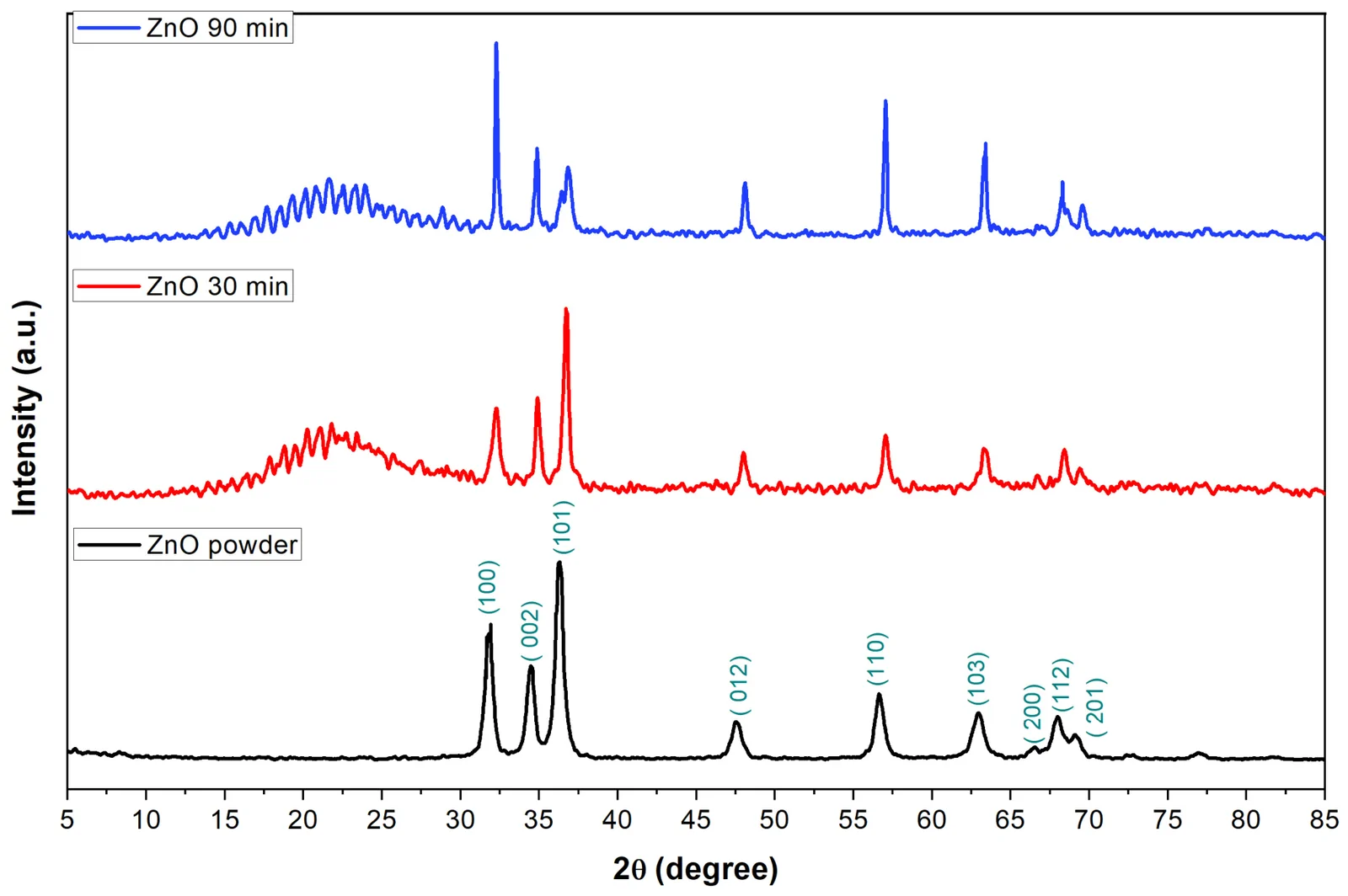
XRD patterns of ZnO powder, ZnO 30 min, and ZnO 90 min samples, indexed to the hexagonal wurtzite ZnO structure (JCPDS no. 01-079-0207).

Figure 5 shows the XRD patterns of the ZnO powder, ZnO 30 min, and ZnO 90 min samples. All three samples are indexed as a hexagonal wurtzite crystal system with space group P6_3_mc and lattice parameters *a* = *b* = 3.2568 Å, *c* = 5.2125 Å (JCPDS no. 01-079-0207) [37]. The prominent diffraction peaks observed at 2*θ* = 31.77°, 34.44°, 36.26°, 47.52°, 56.61°, 62.88°, 66.43°, 67.93°, and 69.07° correspond to the (100), (002), (101), (102), (110), (103), (200), (112), and (201) *hkl* planes of hexagonal ZnO, respectively. No diffraction peaks from any secondary or impurity phase were detected, confirming the phase purity of all samples and indicating that the wurtzite structure is retained throughout the laser post-irradiation process.

The ZnO powder exhibits sharp and intense diffraction peaks, reflecting its high crystallinity. For the ZnO 30 min and ZnO 90 min samples, the same characteristic wurtzite reflections are retained, which indicates that the laser irradiation does not induce a detectable phase transformation. The FWHM of the (101) reflection decreases from 0.55224° for the pristine ZnO powder to 0.36906° after 30 min of irradiation, followed by an increase to 0.44549° after 90 min, indicating a non-monotonic variation in peak width with irradiation time. In addition, a broad hump appears in the low-angle region (15–30° 2*θ*) of the treated samples, which becomes more pronounced with increasing treatment time.

This broad hump is mainly attributed to the amorphous glass substrate onto which the laser-treated colloids were drop-cast and oven-dried, since amorphous silica-based glass is well known to produce a diffuse scattering halo in this angular range. This background is absent in the powder pattern because the powder was measured directly in a sample holder without a glass slide. A secondary contribution may originate from the nanocrystalline and partially amorphous nature of the ZnO nanoparticles generated by pulsed laser ablation in liquid, in which the high quenching rates can retain a fraction of disordered content [38,39].

The average crystallite size (*D*) was estimated from the most intense (101) peak using the Scherrer equation [40]:

*D* = *K λ*/(*β* cos *θ*) (1)

where *K* = 0.9, *λ* = 0.15416 nm is the Cu K*α* wavelength, *β* is the full width at half maximum of the peak, and *θ* is the Bragg angle. The crystallite size increased from 15.14 ± 0.15 nm for the powder to 22.68 ± 0.24 nm after 30 min, followed by a decrease to 18.80 ± 0.48 nm after 90 min. Consistent with the FWHM variation, this non-monotonic behavior indicates an initial increase in the coherent diffraction-domain size followed by a partial decrease upon prolonged irradiation. The emergence of a second diffraction component after 90 min may further indicate increased structural heterogeneity following prolonged laser treatment. The XRD results indicate that laser irradiation initially promotes crystallite growth through photothermal melting and coalescence of the colloidal particles together with recrystallization of disordered fractions [41]. Upon prolonged irradiation for 90 min, the mean crystallite size decreased to 18.80 ± 0.48, accompanied by the emergence of a second diffraction component. This behavior is consistent with a transition from a growth to fragmentation process, in which the accumulated interaction promotes photothermal evaporation and re-nucleation of smaller crystallites, which is attributed to the onset of laser fragmentation [42].

**Figure 6:**
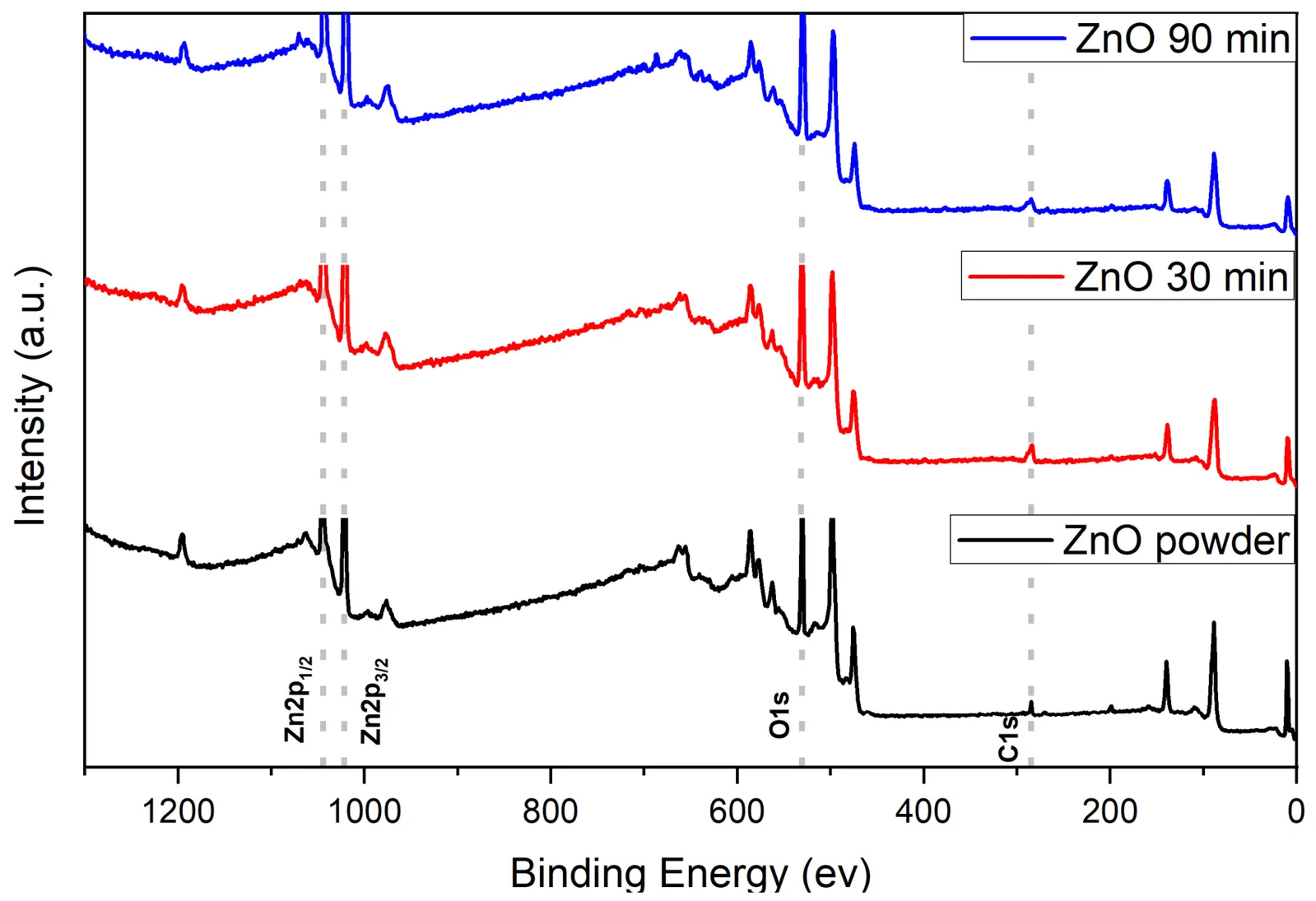
XPS survey spectra of ZnO powder, ZnO 30 min, and ZnO 90 min samples.

Figure 6 shows the XPS wide survey spectra of the three samples. Zn, O, and C signals were detected in all spectra. Minor surface species were also detected in the survey spectra. The pristine ZnO powder contained N and Cl at 0.77 and 3.47 at.%, respectively, while the 30 min sample contained 1.49 at.% N and 2.06 at.% Cl. For the 90 min sample, Cl and F were detected at 3.67 and 4.25 at.%, respectively, whereas N was not detected. The precise origins of these minor species cannot be established from the survey spectra alone. The N signal may be associated with residual synthesis-related or adventitious surface species, while the Cl and F signals are conservatively attributed to trace surface residues or contamination introduced during synthesis, sample handling, or sample preparation.

The surface atomic compositions derived from quantitative analysis of the survey spectra are listed in Table 1. The zinc surface concentration decreases from 42.96 at.% in the powder to 27.36 and 26.59 at.% after 30 and 90 min irradiation, while the oxygen content increases from 43.97 to approximately 49.5 at.%. The O/Zn atomic ratio calculated from the survey spectra increases from 1.02 for the pristine powder to approximately 1.81 and 1.86 after 30 and 90 min irradiation, respectively. Because these values represent the XPS sampled surface and are influenced by surface adsorbates and contamination, the increase should not be interpreted as evidence of further oxidation of Zn. Instead, it indicates a shifting balance between surface-bound oxygen species and lattice, including contributions from surface hydroxyl groups, adsorbed water, and other oxygen-containing surface species. Consistent with this interpretation, the high-resolution Zn 2p spectra indicate that Zn remains predominantly in the Zn^2+^ state after irradiation.

**Figure 7:**
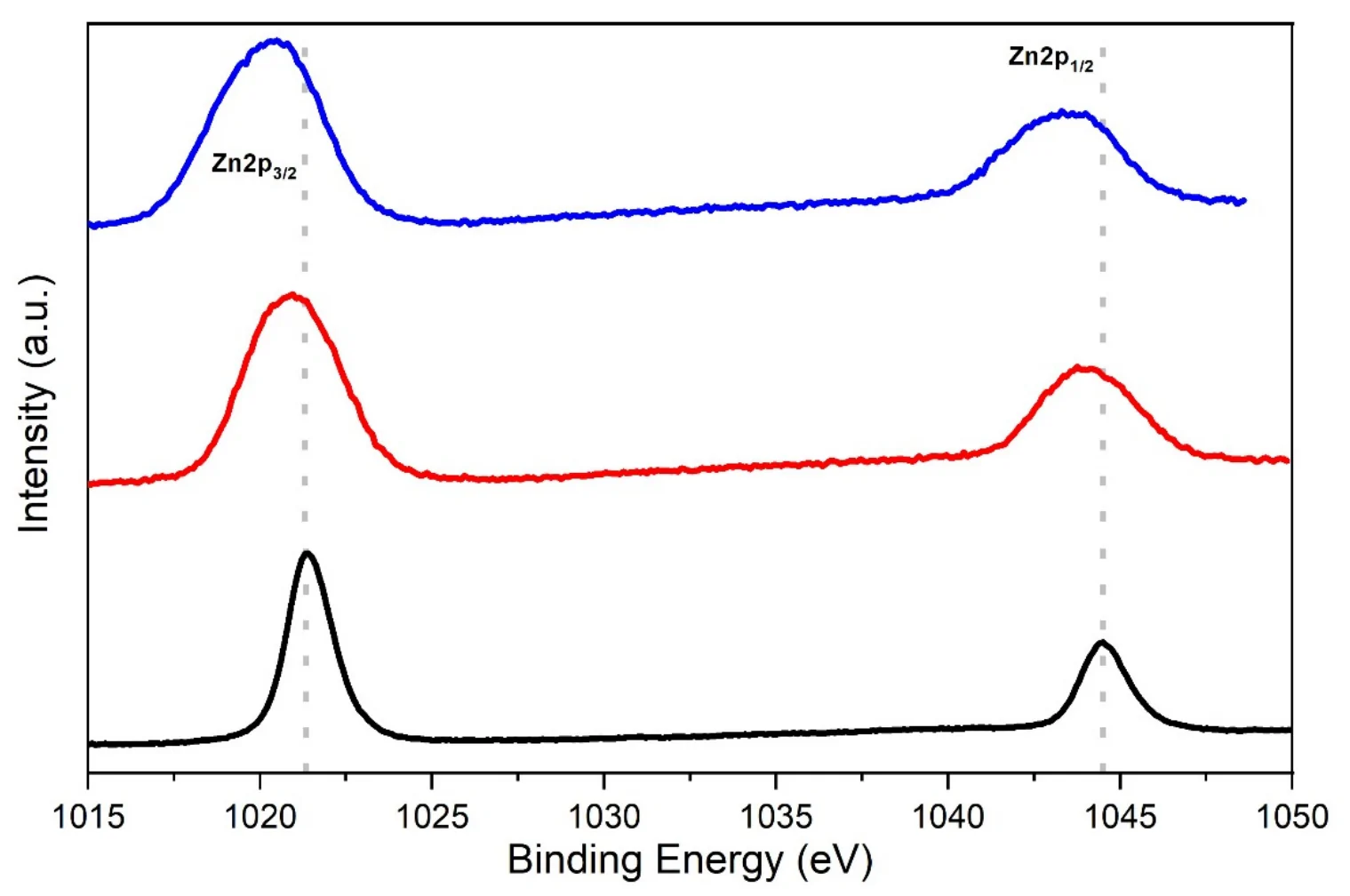
XPS Zn 2p spectra of ZnO powder (black line), ZnO 30 min (red line), and ZnO 90 min (blue line) samples. Dashed lines mark 1021.71 eV (Zn 2p_3_/_2_) and 1044.68 eV (Zn 2p_1_/_2_) of the ZnO powder sample.

Figure 7 shows the Zn 2p spectra for all three samples. The spin orbit doublet Zn 2p_3/2_ and Zn 2p_1/2_ is well resolved in each spectrum. From Figure 7 and Table 1, the Zn 2p_3/2_ binding energies are 1021.71, 1021.11, and 1020.19 eV for the ZnO powder, ZnO 30 min, and ZnO 90 min samples, respectively. These values are comparable to those reported in the literature for ZnO nanoparticles and nanorods [3,43,44].

The Zn 2p_3/2_ peak was fitted to a single Gaussian in all samples. The binding energy difference between Zn 2p_1/2_ and Zn 2p_3/2_ is ~23 eV for all three samples. These results confirm that zinc retains its +2 oxidation state throughout laser processing. A progressive broadening of the Zn 2p_3/2_ peak is observed, with FWHM increasing from 2.39 eV (e.g., ZnO Powder) to 3.73 eV (e.g., ZnO 90 min), reflecting an increasingly disordered surface environment with laser irradiation.

**Figure 8:**
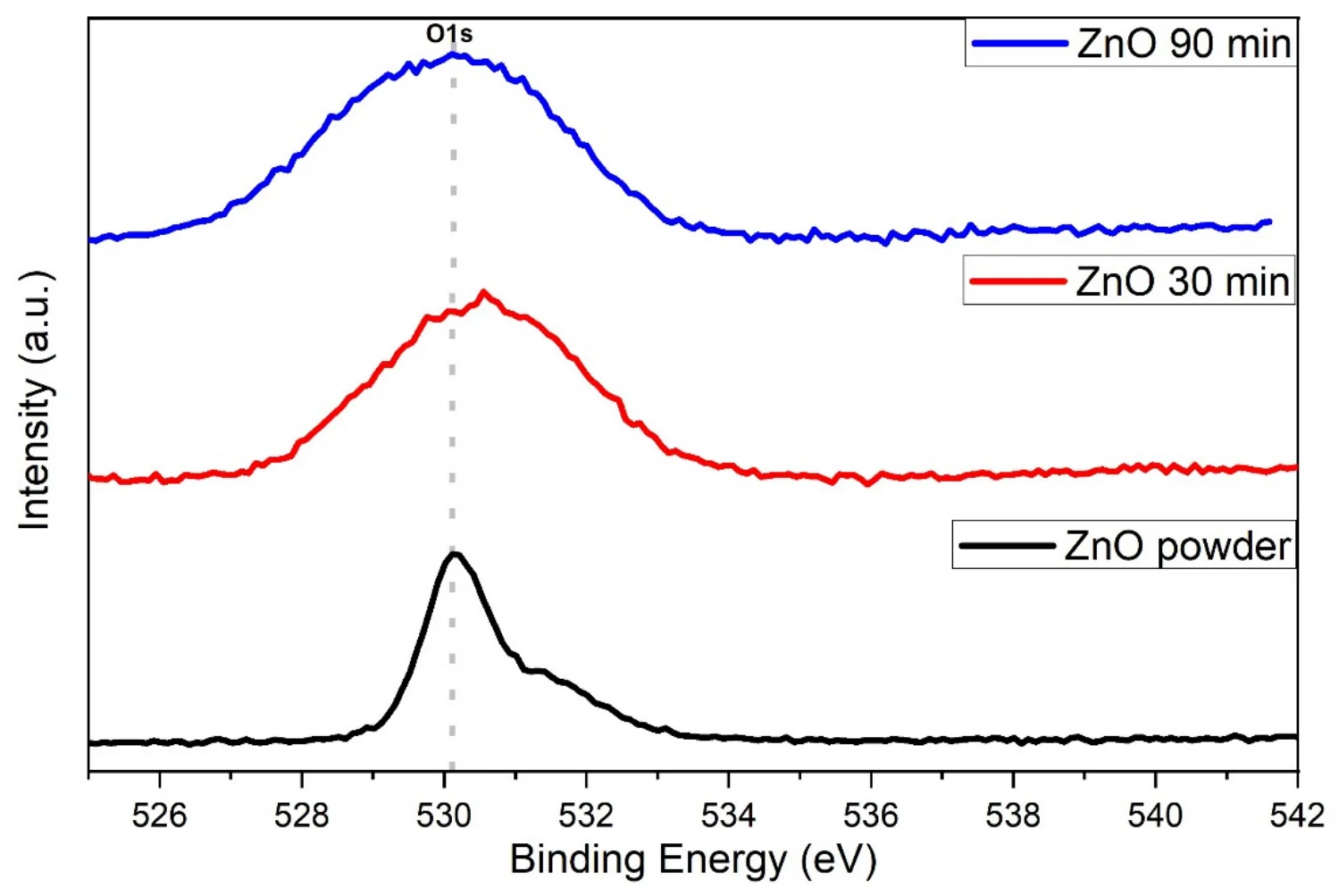
O 1s comparison spectra of ZnO powder, ZnO 30 min, and ZnO 90 min samples. Dashed line at 530.11 eV marks the O 1s peak maximum of the ZnO powder sample.

**Figure 9:**
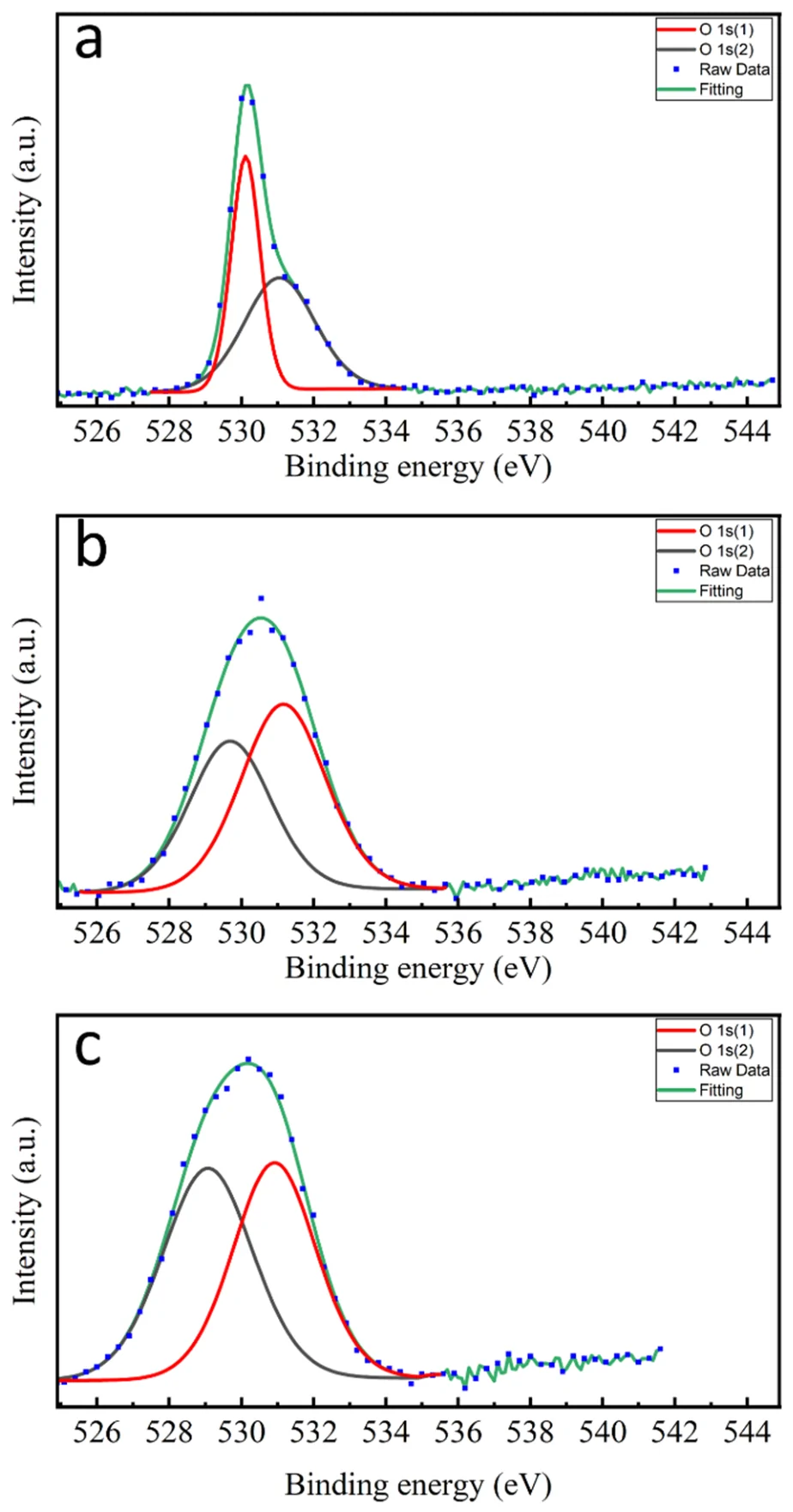
High-resolution O 1s XPS spectra with deconvolution fits of (**a**) ZnO powder, (**b**) ZnO 30 min, and (**c**) ZnO 90 min samples.

Figure 8 shows the O 1s comparison spectra and Figure 9 displays the individual deconvolution fits for each sample. The dashed reference line in Figure 8 is placed at 530.11 eV, corresponding to the O 1s peak maximum of the ZnO powder high-resolution spectrum.

Each O 1s spectrum was fitted with a Shirley background and two Gaussian–Lorentzian components, as shown in Figure 9 and summarized in Table 1. For the pristine ZnO powder, the O 1s core level shows two components. Peak (1) at 530.11 eV is assigned to O^2−^ ions in the Zn–O bonding of the wurtzite structure of ZnO [3,45]. Peak (2) at 531.01 eV is related to OH groups adsorbed onto the ZnO surface [3,46]. These values are in good agreement with those reported in the literature [3,45,46]. In accordance with recent theoretical calculations, the O 1s (2) component is not assigned to oxygen vacancies, since a vacant oxygen site emits no photoelectrons and vacancies have a negligible effect on the O 1s binding energies of neighboring oxygen atoms [47].

For the 30 min sample, peaks (1) and (2) are located at 529.65 and 531.15 eV, respectively. The increase in the O 1s (2) fraction from 54.0% (pristine) to 56.1% (30 min) indicates that laser irradiation promotes hydroxylation at freshly generated surface defect sites. At 90 min, the two components are located at 529.10 and 530.90 eV, with relative areas of 51.5% and 48.5%, respectively. The recovery of the lattice oxygen fraction at 90 min, exceeding the pristine value, is consistent with the formation of more compact, nanoparticles observed by TEM. The ratio O 1s(1)/O 1s(2) evolves from 0.85 for the pristine powder to 0.78 after 30 min and 1.06 after 90 min, tracking surface disorder non-monotonically with laser irradiation. The increase in the survey-derived O/Zn ratio from ~1.02 to ~1.86 indicates an increase in the relative oxygen content of the XPS sampled surface with irradiation. This change is not interpreted as a change in the Zn oxidation state, but rather, in conjunction with the O 1s analysis, as reflecting changes in the relative contributions of lattice and surface oxygen-containing species.

## 4. Conclusions

In summary, the morphology and surface chemical composition of ZnO nanostructures were successfully engineered through controlled nanosecond pulsed laser post-irradiation of as-prepared ZnO nanorods in deionized water. The study demonstrated a progressive transformation from strongly agglomerated, faceted nanorods to a mixed rod and particle morphology after 30 min of irradiation and finally to well-dispersed, nearly spherical nanoparticles with diameters of approximately 12–48 nm after 90 min, driven by a photothermal melting and fragmentation mechanism enabled by the above-band-gap photon energy of the 355 nm laser pulses. XRD confirmed that the hexagonal wurtzite phase was preserved at all stages of the treatment without any secondary or impurity phases, while the mean crystallite size increased from 15.14 nm for the powder to 18.8 nm after 90 min as a result of photothermal fusion of the crystallites. XPS revealed that zinc retains its +2 oxidation state throughout the laser processing, whereas the surface O/Zn ratio and the relative fractions of lattice oxygen and surface hydroxyl species evolve systematically with irradiation time, indicating that the laser treatment simultaneously reshapes the nanostructures and modifies their surface chemistry. These results establish the laser post-irradiation duration as a simple, additive-free control parameter for tuning both the morphology and the surface chemical state of ZnO nanostructures. Such controlled tuning is of potential interest for applications including photocatalysis, sensing, and biomedicine, where morphology and surface chemistry are known to influence performance.

## Figures and Tables

**Table 1:** XPS analysis of ZnO powder, ZnO 30 min, and ZnO 90 min laser-irradiated samples.

Peak	Position BE (eV) ±0.10	FWHM (eV) ±0.20	Raw Area (cps·eV)	Atomic %
ZnO Powder				
Zn 2p_3_/_2_	1021.71	2.39	3,620,710	28.64
Zn 2p_1_/_2_	1044.68	2.39	1,810,355	14.32
O 1s (1) Zn–O	530.11	0.80	402,221	20.24
O 1s (2) Zn–OH	531.01	2.30	471,758	23.73
C 1s	284.80	2.26	68,831	8.84
N 1s	399.00	3.60	10,112	0.77
Cl 2p	199.49	1.82	64,234	3.47
ZnO 30 min				
Zn 2p_3_/_2_	1021.11	3.44	1,712,836	18.24
Zn 2p_1_/_2_	1044.08	3.44	856,418	9.12
O 1s (1) Zn–O	529.65	2.50	321,389	21.76
O 1s (2) Zn–OH	531.15	2.60	411,353	27.84
C 1s	284.80	5.13	112,808	19.50
N 1s	401.32	1.70	14,578	1.49
Cl 2p	201.24	4.93	28,352	2.06
ZnO 90 min				
Zn 2p_3_/_2_	1020.19	3.73	1,490,027	17.73
Zn 2p_1_/_2_	1043.16	3.73	745,014	8.86
O 1s (1) Zn–O	529.10	2.80	336,807	25.47
O 1s (2) Zn–OH	530.90	2.60	316,797	23.95
C 1s	284.80	8.05	83,172	16.06
F 1s	690.08	3.05	76,306	4.25
Cl 2p	202.00	5.71	45,256	3.67

## Data Availability

Data are available upon request.
